# A rare case of scleral buckle infection with Curvularia species

**DOI:** 10.1186/s12886-018-0695-4

**Published:** 2018-02-09

**Authors:** Shalini Singh, Ankita Shrivastav, Manisha Agarwal, Arpan Gandhi, Rahul Mayor, Lagan Paul

**Affiliations:** Vitreoretina services, Dr. Shroff’s Charity Eye Hospital, 5027, Kedarnath Road, Daryaganj, New Delhi, 110002 India

**Keywords:** Buckle infection, Dematiaceous fungus

## Abstract

**Background:**

Scleral buckling is an established modality of treating retinal detachment. Being an external implant the buckle may be prone to infections. We report such a case with a delayed presentation and a rare etiology.

**Case presentation:**

A 45 year old male presented with redness, foreign body sensation and discharge for one month in his right eye. The patient had undergone a retinal detachment surgery elsewhere 14 years back without any visual gain. Right eye demonstrated no perception of light and the best corrected visual acuity in the left eye was 6/6, N6. On downgaze an exposed and anteriorly displaced scleral buckle was identified with black deposits and mucopurulent material overlying the buckle. Scleral buckle removal was done. On microbiological examination Curvularia species was identified. Successful treatment with antifungals was done.

**Conclusions:**

Scleral buckle infection with dematiaceous fungi is a rare occurrence. To the best of our knowledge this is the first case report describing a buckle infection caused by the curvularia species.

## Background

Scleral buckling has been an established and effective surgical method for treating retinal detachments since over 60 years [1]. In the recent years pars plana vitrectomy has become more popular, however, scleral buckling has several indications as the primary procedure of choice in a rhegmatogenous retinal detachment. Being an external implant the scleral buckle is prone to get infected and the infection may set in even years after the primary procedure. The commonest organisms reportedly causing a scleral buckle infection are gram positive cocci [2–4], however, in rare cases a fungal infection may set in especially in an immunocompromised host. We report a case of buckle infection due to Curvularia species in an immunocompetent patient. To the best of our knowledge this is the first report of a case describing buckle infection due to Curvularia species.

## Case report

**Fig. 1:**
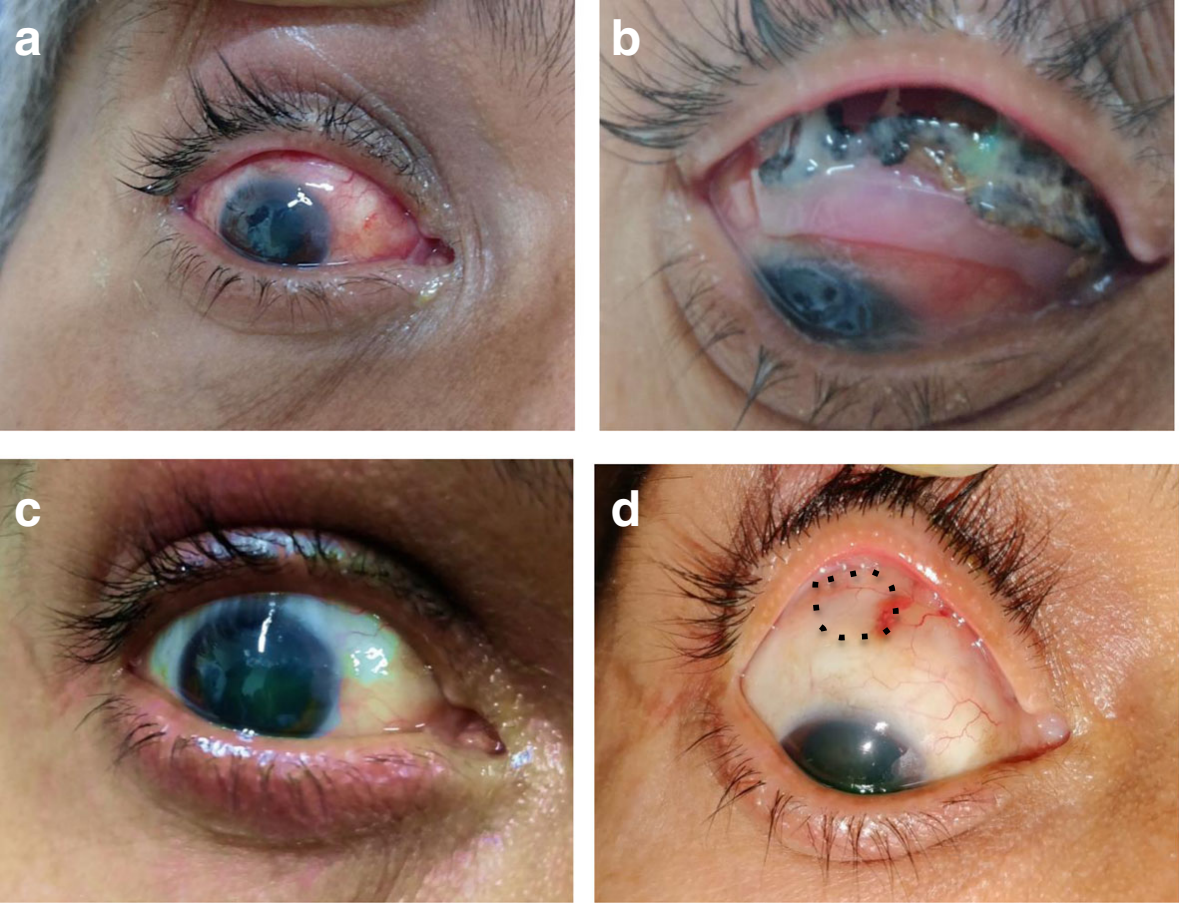
Conjunctival congestion (**a**) and necrotic black discharge (**b**) seen prior to buckle removal. Clear conjunctiva (**c**) seen after buckle removal with areas of scleral thinning (**d**), dashed lines

**Fig. 2:**
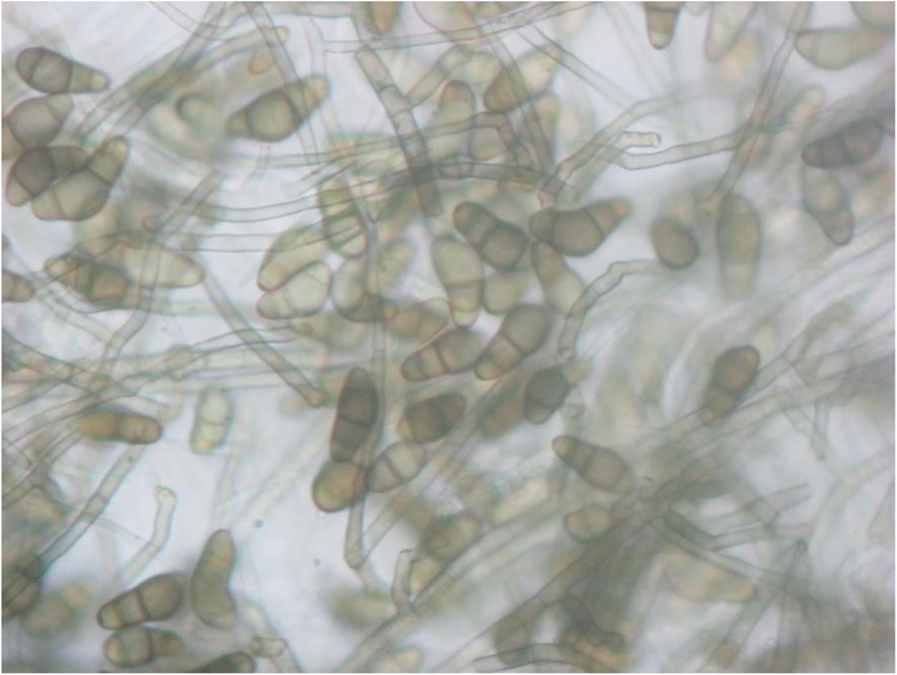
Curvularia species identified on culture medium showing pigmented hyphae and conidia

A 45 year old male presented to us with complaints of redness, discharge and foreign body sensation in his right eye since one month. He had undergone a scleral buckling procedure elsewhere 14 years back of which he had no records. There was no visual gain post-operatively. He had no history of trauma and is a healthy patient without any systemic diseases. On examination, he had no perception of light in the right eye. Mucopurulent discharge was present and conjunctival congestion was noted. A circumferentially placed and anteriorly displaced exposed solid scleral buckle explant was noted with black coloured deposit over the buckle in the superior quadrant (Fig. 1(a), (b)). Anterior chamber was deep and quiet and lens was clear. Fundus examination revealed a visible buckle indentation superiorly and an inferior retinal detachment with disc pallor. No visible inflammatory reaction was noted internally. The left eye had a best corrected visual acuity of 6/6, N_6_ with a normal anterior segment and fundus picture. A diagnosis of exposed and anteriorly displaced infected scleral buckle was made. Removal of the infected buckle was planned. The conjunctiva was dissected and the buckle along with sutures was removed and sent for microbiological evaluation. The eye was cleared of the necrotic debris and discharge. A thorough subconjunctival wash was done with antibiotic (gentamycin) and antifungal agents (voriconazole) and the conjunctival peritomy was closed. The patient was started on empirical treatment with oral ciprofloxacin 750 mg twice a day, oral fluconazone 150 mg twice a day, eye drop moxifloxacin hourly, eye drop voriconazole hourly, eye drop atropine 3 times a day and eye drop carboxymethylcelluose 0.5% 6 times a day in the right eye. Both blood agar and Sabouraud’s dextrose agar grew blackish colonies with irregular boundaries. On microbiological evaluation of the growth, pigmented hyphae were noted along with the presence of conidiophores and conidia and a diagnosis of Curvularia species was made (Fig. 2). The patient received oral ciprofloxacin for one week and fluconazole for three weeks. The patient was followed up closely and at 1 month after buckle removal he had no conjunctival congestion, the superotemporal sclera appeared thinned out (Fig. 1 (c), (d)). Topical medications were stopped. We presume that the inferior retinal detachment was exudative in nature as on subsequent follow-up at 2 months it was seen to have settled and no break was found.

## Discussion

The most common indications for removal of a scleral buckle are buckle infection and buckle exposure [2, 5], with their incidence ranging from 0.2–5.6% after a scleral buckling procedure [6].

The most commonly isolated organism has been reported to be coagulase negative staphylococcus, mainly *Staphylococcus epidermidis* [2, 4, 7]. However, there have also been reports of buckle infections caused my atypical mycobacteria, corynebacteria and fungi [8]. Immunocompromised hosts are particularly susceptible to fungal infections and the commonest fungus causing a buckle infection is reported to be Aspergillus species [4, 7, 8].

Dematiaceous fungi are ubiquitous in nature and are common saprophytes on plant material and in soil, developing dark colonies. In the eye they most frequently cause a corneal infection. Some of the dematiaceous fungi known to cause ocular infection are Alternaria, Curvularia, Bipolaris, Exserohilum and Coelomycetes species [9].

It is extremely rare to find a buckle infection caused by a dematiaceous fungus. Bakri et al. have reported a buckle infection caused by the dematiaceous fungus Alternaria species [10].

In our case, a young healthy male developed a dematiaceous fungal infection with Curvularia species and presented with the infection 14 years after the scleral buckle procedure. Buckle infections may present years after the primary procedure and it is important for clinicians to be aware of such delayed presentations which may occur even in immunocompetent individuals.

## Conclusion

To the best of our knowledge this is the first case report describing the morphological and microbiological findings in a buckle infection caused by Curvularia species. This case highlights the importance of a strong suspicion of fungal etiology based on the clinical presentation and the need for urgent management. Clinicians need to be aware of this entity and not disregard its possibility in an immunocompetent individual.
